# Supplementary material for: Neuropsychological Predictors of Fatigue in Post-COVID Syndrome
Source: J Clin Med. 2022 Jul 4;11(13):3886. doi: 10.3390/jcm11133886 (PMC9267301; doi:10.3390/jcm11133886)
Supplement: Supplementary file 1 [file jcm-11-03886-s001.zip › jcm-1803402-supplementary.pdf]

**SUPPLEMENTARY TABLE S1.** Description of neuropsychological tests and scores.	
**NEURONORMA battery (NN)**	
**Digit span**	**Forwards and Backwards:** This task involves a measure of verbal attention and memory. The digit span requires repeating sequences of digits of increasing length forward and then in reverse order.<br>The main variable, in both cases, is the length of the longest series correctly returned. Two variables were analyzed, one for direct digits, with a range from 0 to 9, and one for inverse digits, with a range from 0 to 8.
**Corsi Blocks Forwards**	**Forwards and Backwards:** It consists of nine cubes fastened in a random order in a board. Each time the examiner taps the blocks into a prearranged sequence, the patient must attempt to copy this pattern. This is both an attention and memory task. Backward Span involves mental tracking with visual processes and includes a Working Memory component.
**Symbol Digit Modalities Test (SDMT)**	SDMT measures divided attention, visual scanning, visual tracking, perceptual speed, motor speed, and memory. The subject is required to scan the key and write down the number corresponding to each design as rapidly as possible in 90 seconds. There is a practice period (10 boxes).<br>Main variable: The number of correct responses is recorded. The maximum score is 110.
**Boston Naming Test (BNT)**	A measure of assess visual naming ability. The main variable is obtained from the sum of items called without help and those correct after the semantic clue, with a range from 0 to 60.
**Judgment of Line Orientation Rey Figure (JLO)**	A measure of visuoperceptive ability and spatial orientation. The version used is composed of 20 items, which must be matched to the model based on their inclination. The score ranges from 0 to 20.
**Rey-Osterrieth Complex Figure (ROCF)**	The objective of this task is to assess visual perception, visual–spatial constructional ability, and visual memory. It also measures a series of cognitive capacities including planning and problem-solving strategies.<br>Main variables**: ROCF Copy, ROCF Time**, two recall trials: one at 3 minutes and the other at 30 minutes (**ROCF 3 min and ROCF 30 min**) and a recognition subtest (**ROCF recognition**).
**Free and Cued Selective Reminding Test (FCSRT)**	The FCSRT is a measures verbal learning and memory in which 16 words presented in written form must be recalled.<br>Main variables: **Free recall trial 1** (range 0 to 16), **FCSRT total free recall** (range 0 to 48), **FCSRT total recall** (sum of free and facilitated, range 0 to 48), **FCSRT delayed free recall** and **FCSRT delayed total recall** (range 0 to 16 in both variables).

**Verbal fluencies**	Verbal fluency tasks supply data on verbal productivity, semantic memory, language, and executive function. Consists of three task: **Verbal fluency animals:** The objective is to generate as many words as possible for the semantic category "animals"; **Verbal fluency P and M:** The objective of task is to generate as many words as possible beginning with P and M.<br>Sixty seconds were allowed for each category.
**Stroop Color-Word Interference Test (STROOP)**	This test is a measure of cognitive flexibility, selective attention, cognitive inhibition, and information processing speed. It consists of 3 parts: **Stroop A** (a list is shown with the name of three randomly repeated colors), **Stroop B** (a list is shown with letters "X" printed in the ink of those three colors and the subject must identify and name the colors), **Stroop C** (a list is shown in which the names of the colors are written in ink of a color incongruent with their name and the subject must say the color of the ink, inhibiting reading). The three parts are analyzed, consisting of the number of items performed in the 45 seconds allowed for each of the parts.
**Visual Object and Space Perception Battery (VOSP)**	**VOSP object decision:** This test consists of 20 arrays, each of which displays one real two-dimensional object together with three distracter items. The number of correct choices (maximum 20) is recorded; **VOSP progressive silhouettes**: This task consists of two series of stimulus cards each comprised of 10 silhouette drawings. Each successive drawing progressively reveals more details of the object. The subject is required to identify the object as early as possible. The number of trials required to identify each object is totaled and recorded as the score. Maximum score is 20 ; **VOSP position discrimination:** This test consists of 20 cards. Each card contains two adjacent squares containing dots: one with a black dot printed exactly in the center and one with the black dot just off-center. The participant has to decide which square has the dot in the center. The number of correct choices is recorded as the score (maximum score is 20); **VOSP number location:** This test consists of 10 cards. Each card shows two squares, one above the other with a small gap between them. The top square contains randomly placed numbers (1–9) and the bottom square contains a single black dot corresponding to the position of one of the numbers. The task is to identify the number that corresponds with the position of the dot. The total number of correct responses is recorded (maximum score is 10).

Vienna Test System Battery (VTS)		
**COGBAT: Cognitive Basic Assessment**	**Trail Making Test (Langensteinbach version), parts A and B (S1 form).**	**Main variables: Working time – Part A**: a measure of the respondent's cognitive processing speed. This variable includes the total working time of all items. Working time is defined as the time between the correct clicking or tapping of two consecutive numbers; **Working time – Part B:** a measure of the ability to shift at will between different reference systems when this is required. This variable includes the total working time of all items. Working time is defined as the time between the correct clicking or tapping of two consecutive numbers or letters.
		**Errors in Parts A and B**: This variable gives the number of errors in Part A and Part B; **Difference score (B-A):** This variable is the same as the difference score: it is the difference between the working times in Part B and Part A and Quotient B/A1 This variable is the same as the ratio score: it is the quotient of the working times in Part B and Part A; **Working time – Part A (corrected)** : This variable is the sum of the corrected working times in Part A. The purpose of this calculation is to take errors into account in the assessment of working time. The corrected working time is the time between the last (correct or incorrect) clicking or tapping of a number and the correct tapping of a number and **Working time – Part B (corrected):** This variable is the sum of the corrected working times in Part B. It is calculated in the same way as the corrected working time for Part A.
	**The Figural Memory Test (FGT) (S11 form)**	**Learning total**: the sum of correct entries in learning runs 1 to 5; **Delayed free reproduction I Short-term:** Short-term delayed recall takes place after a brief break of five minutes following the last repetition of the item list, without the list being presented to the respondent again. The variable "Delayed free reproduction I" records the number of figures entered correctly during this run; **Delayed free**

		**reproduction II:** this variable measures the retention of figural material, this time after a longer break of 30 minutes. The variable “Delayed free reproduction II” records the number of figures entered correctly during this run; **Delayed free reproduction II:** this variable measures the retention of figural material, this time after a longer break of 30 minutes. The variable “Delayed free reproduction II” records the number of figures entered correctly during this run; **Correct – recognition run:** In the recognition run either 18 figures are presented. For each one the respondent must state whether it was among the figures that were learned earlier. The variable “Correct – recognition run” gives the number of correct answers in this recognition run; **Correct – learning runs 1 – 5:** these five variables record the number of correctly entered figures in the learning run concerned; **Errors – learning runs 1 – 5 and Errors – delayed recognition runs:** any reproduction of an item that is not faithful to the original (rotation/reflection, incorrect positioning of any detail) and any repetition of a figure that has already been entered in the same reproduction run is classed as an “error; **Errors – Recognition run:** errors in the recognition run arise from incorrect assessment of an item by the respondent.
	**Response inhibition (S13)**	**Main variable: Number of commission errors:** this variable describes how frequently inhibition of no-go stimuli was unsuccessful.
		**Subsidiary variables: Number of omission errors:** this variable reports the number of omitted reactions to go stimuli**; Sensitivity index d':** a measure of overall performance on the task; it takes account of both commission errors and omission errors; **Mean reaction time:** this variable is calculated as the mean reaction time for correctly processed go stimuli; **Mean reaction**

		**time:** this variable is calculated as the mean reaction time for correctly processed go stimuli.
	**N-Back verbal (S1 form)**	**Main variable: Correct:** The number of correct responses to a target stimulus (=total hits).
		**Subsidiary variables: Omitted:** The number of omitted responses to a target stimulus (=total missed stimuli); **Incorrect:** The number of false positive responses (=total false alarms). This variable describes the number of false alarm; **Mean time "correct":** The mean reaction time for correct responses (mean response time "hits"); **Mean time "incorrect":** The mean reaction time for incorrect responses (mean response time "false alarms"); **Working time:** This variable indicates the working time spent on the test in minutes and seconds.
	**Tower of London (Freiburg version) (TOL, S1)**	**Main variable: Planning ability number:** Number of the four- to six-move items solved in the minimum number of moves.
		**Subsidiary variables: Number of correctly solved items:** Number of items in which the objective was achieved (in any number of moves); **Time limits exceeded:** Number of items terminated because the pre-set time limit was exceeded; **Reversed decisions:** "Undoing" a ball that has already been moved by clicking on it again; **Selection of a blocked ball:** Error or infringement of a rule by picking up a ball that is blocked by one above it; **Selection of a blocked rod:** Error or infringement of a rule by placing a ball on a rod that is already full**; Selection of an impossible position:** Error or infringement of a rule by picking up or placing a ball by clicking outside the area defined by the tower configuration of the start state; **Median planning time:** Median planning times, reported separately for four-, five- and six- move problems. Calculation is based only on items in which the goal state was achieved; **Median execution time:** Median execution times, reported separately for four-, five- and six- move

		problems. Calculation is based only on items in which the goal state was achieved.
	**MOUSE**	**Mouse control:** this variable summarizes the respondent's speed and accuracy; **Number of correct reactions:** the number of squares that are clicked within the time limit; **Speed:** this variable is the median of the working times for all squares; **Precision:** calculation of this variable involves measuring for each square where the cursor was when the square appeared and when it disappeared.
	**FLEI**	FLEI is a questionnaire consisting of 35 statements, which must be rated in turn. Answers are entered on a five-point rating scale (never – rarely – sometimes – often – very often). These variables describe the subjective ability reported by the respondent in five areas: **Mental ability, Attention, Memory, Executive functions and Control scale.**<br>The raw score on the mental ability scale is the sum of the respondent's raw scores on the scales Attention, Memory and Executive functions.
**WAF: Perception and Attention Function Battery Assessment**		
	**WAF: Alertness (S11)**	Encompasses the construct of intrinsic alertness: the state of ongoing general wakefulness and readiness to react. **Main variable(s): Intrinsic alertness (visual) ; Intrinsic alertness (visual) – retesting ; Cross-modal phasic alertness (visual); Unimodal phasic alertness (visual); Intrinsic alertness (auditory); Intrinsic alertness (auditory) – retesting ; Cross-modal phasic alertness (auditory); Unimodal phasic alertness (auditory).**
		**Subsidiary variable(s): Median reaction time:** this is the median of all reaction times; **Dispersion of reaction time:** this is the exponent of the standard deviation of logarithmic reaction times ; **Number of missed reactions:** this is the number of stimuli to which no response was made within 1500 ms; **Number of**

		**false alarms:** this is the number of times a response key was pressed when no stimulus had been presented; **Number of premature reactions:** in the subtests for phasic alertness, reactions which occur between the cue and the actual stimulus are classed as “premature reactions”; **Resilience to fatigue**: these variables are the difference between intrinsic alertness and intrinsic alertness – retesting.
	**Divided attention, cross-modal (visual/ auditory) (S1)**	Measures divided attention: the ability to direct one’s attention towards several information channels simultaneously. **Main variable(s): Unimodal divided attention (visual) and Cross-modal divided attention (visual/auditory).**
		**Subsidiary variable(s): Median reaction time:** this is the median of all reaction times ; **Dispersion of reaction time:** this is the exponent of the standard deviation of logarithmic reaction times; **Number of missed reactions:** this is the number of stimuli to which no reaction occurred within 1500 ms (long and short forms) or 2000 ms (simple short form); **Number of false alarms:** this is the number of times a response key was pressed when no stimulus had been presented ; **Number of correct reactions:** this variable gives the number of correct reactions to relevant stimuli.
	**Vigilance and sustained attention dimension**	Measures the ability to react to monotonous signals over a long period of time with low proportion of relevant stimuli. **Main variable(s): Vigilance (visual); Vigilance (auditory); Sustained attention (visual); Sustained attention (auditory).**
		**Subsidiary variable(s): Median reaction time:** this is the median of all reaction times; **Dispersion of reaction time:** this is the logarithmic standard deviation of the reaction times**; Number of missed reactions:** this is the number of stimuli to which no response was made within 1000 ms**; Number of false alarms:** this

		variable gives the number of reactions to false or non-existent stimuli**.**
	**Focused attention dimension**	**Main variable(s): Unimodal focused attention (visual); Unimodal focused attention (auditory) ; Cross-modal focused attention (visual/auditory).**
		**Median reaction time:** this is the median of all reaction times; **Dispersion of reaction time:** this is the logarithmic standard deviation of the reaction times**; Number of missed reactions:** this is the number of stimuli to which no response was made within 1500 ms. **; Number of false alarms:** this variable gives the number of reactions to false or non-existent stimuli.
	**Selective attention dimension**	Measures performance in a choice reaction task. **Main variable(s): Unimodal selective attention (visual); Unimodal selective attention (auditory) ; Cross-modal selective attention (visual/auditory :** a main variable with the name of the ability it measures is available for each subtest from the Focused attention dimension.
		**Subsidiary variable(s): Median reaction time:** this is the median of all reaction times; **Dispersion of reaction time:** this is the exponent of the standard deviation of logarithmic reaction times. **Number of missed reactions:** this is the number of stimuli to which no response was made within 1500 ms; **Number of false alarms:** this is the number of times a response key was pressed when no stimulus had been presented; **Number of correct reactions:** this variable gives the number of correct reactions to relevant stimuli.
	**Spatial attention dimension**	Automated spatial attention: Assesses the ability to shift someone's attention from one spatial focus to another; Assesses visual neglect: an attention disorder where the sufferer tends to be unaware of stimuli on the side contralateral to the lesion. **Main variable(s): Controlled spatial attention; Automated spatial attention; Right-side neglect; Left-side neglect.**

		**Subsidiary variables – controlled and automated spatial attention: Number of false alarms:** this is the number of times a response key was pressed when no stimulus had been presented. The following variables are available separately for unannounced, correctly announced and incorrectly announced stimuli; **Controlled spatial attention**: these variables are a logarithmic mean of the individual reaction times to unannounced stimuli; **Automated spatial attention:** these variables are a logarithmic mean of the individual reaction times to unannounced stimuli; **Median reaction time**: this is the median of all reaction times; **Dispersion of reaction time: this** is the exponent of the standard deviation of logarithmic reaction times.; **Number of missed reactions:** this is the number of stimuli to which no response was made within 1500 ms.<br>The following variables are available separately with long or short stimulus onset asynchrony (SOA = delay between the cue and the stimulus): **Controlled spatial attention:** these variables are a logarithmic mean of the individual reaction times to unannounced stimuli ; **Automated spatial attention:** these variables are a logarithmic mean of the individual reaction times to unannounced stimuli; **Median reaction time:** this is the median of all reaction times; **Dispersion of reaction time:** this is the exponent of the standard deviation of logarithmic reaction times**.**<br>**Subsidiary variable(s) – Neglect:** The following variables are available separately for stimuli which are presented on the right or on the left side; Median **reaction time:** this is the median of all reaction times; **Dispersion of reaction time:** this is the exponent of the standard deviation of logarithmic reaction times; **Number of missed reactions:** this is the number of stimuli to which no response was made within 3000 ms; Number of errors: this variable gives the number of errors.

		**The following variables are available for stimuli which were presented on both sides: Neglect, bilateral:** these variables are a logarithmic mean of the individual reaction times to unannounced stimuli; **Median reaction time, bilateral:** this is the median of all reaction times; **Dispersion of reaction time, bilateral**: this is the exponent of the standard deviation of logarithmic reaction times; **Number of missed reactions, bilateral:** this is the number of stimuli to which no response was made within 3000 m; **Number of missed reactions, bilateral – right/left**: these two variables are the number of stimuli presented on both sides to which only a reaction occurred on the left/right, but not on both sides. **Additional information and automated spatial attention: The following variables are available depending on the direction in which the tip of the triangle is pointing. The association is as follows: 0° = top, 90° = triangle pointing right; 180° =triangle pointing down; 270° =triangle pointing left; Controlled spatial attention:** these variables are a logarithmic mean of the individual reaction times to unannounced stimuli**; Automated spatial attention:** these variables are a logarithmic mean of the individual reaction times to unannounced stimuli**; Median reaction time:** this is the median of all reaction times**; Dispersion of reaction time:** this is the exponent of the standard deviation of logarithmic reaction times**; Number of missed reactions:** this is the number of stimuli to which no response was made within 1500 ms.
	**Smooth pursuit eye movements dimension**	Assesses the ability to discriminate moving objects for which the eyes must match the object speed as accurately as possible. **Main variable: Smooth pursuit eye movements:** this variable is available for both subtests from the smooth pursuit eye movements dimension.

		**Subsidiary variable(s): Median reaction time:** this is the median of all reaction times; **Dispersion of reaction time: this** is the logarithmic standard deviation of the reaction times; Number of correct reactions: this is the number of correct reactions; **Number of missed reactions:** this is the number of stimuli to which no response was made within 1000 ms; **Number of false alarms:** this variable gives the number of reactions to false or non-existent stimuli; **Additional information:** the variables Correct reaction and Number of missed reactions are available according to the quadrant of the screen, respectively. In addition, the variables Smooth pursuit eye movements, Number of correct reactions and Number of missed reactions are also available by the direction of movement (upwards, downwards, to the right and to the left).
	**Visual scanning dimension:**	Two subtests on visual scanning show a 6x6 matrix of simple visual stimuli on the screen**.** **Main variable(s): Visual scanning (accuracy):** a measure from signal detection theory that describes the ability to differentiate between target stimuli (signals) and distractors (noise) and **Visual scanning (speed).**
		**Subsidiary variable(s): Median reaction time:** this is the median of all reaction times. These reaction times are also cleared for positions before calculating the variable; **Dispersion of reaction time:** this is the logarithmic standard deviation of the reaction times. **Number of hits:** this is the number of correct positive responses. **Number of true rejections:** this is the number of correct negative responses. **Number of missed reaction:** this is the number of false negative responses; **Number of false alarms**: this is the number of false positive responses.
**Cognitrone (S11 form)**		**Main variable: Mean time "correct rejections" (sec):** the average time taken by the respondent to decide that the comparison figure is not identical to any of the reference figures**.**

		**Subsidiary variables: Total "hits":** the number of responses in accordance with the rules – i.e. the number of instances in which the figures were identical and the respondent pressed the green button; **Total "correct rejections":** the number of instances in which the figures were not identical and the respondent pressed the red button; **Mean time "hits" (sec.):** the average time taken by the respondent to decide that the comparison figure is identical with any of the reference figures.
**RT: The Reaction Test**		**Main variables: Reaction speed:** When the rest button is used the reaction speed is the time between the appearance of the relevant stimulus and the moment the finger leaves the rest button. The variable Reaction speed is the mean of the Box-Cox transformed reaction times; **Motor speed**: is the time that elapses between the moment the finger leaves the rest button and the time the black reaction button is pressed in response to a required stimulus. The mean motor speed is the mean of the Box-Cox transformed reaction motor time.<br>Subsidiary variables: **Measure of dispersion reaction speed:** is the standard deviation of the Box-Cox transformed reaction times;<br>**Measure of dispersion motor speed:** is the standard deviation of the Box Cox-transformed motor times.
		**Additional results: Number of correct reactions:** occurs when the respondent releases the rest button and then presses the black reaction button in response to relevant stimuli; **Number of missed reactions:** occurs if the respondent's finger does not leave the rest button in response to a relevant signal; **Number of incomplete reactions:** occurs if the respondent removes his finger from the rest button when a relevant stimulus is presented but does not then press the black reaction button; **Number of false alarms:** A false alarm occurs if the

		respondent removes his finger from the rest button and then presses the black reaction button in response to irrelevant stimuli.
**DT: DETERMINATION TEST**		**Main variable: "Correct":** describes the number of accurate (= on-time plus delayed) reactions. Only one correct reaction per stimulus is counted even if the button is pressed repeatedly.
		**Subsidiary variables: Incorrect:** each inappropriate reaction to a stimulus is regarded as an incorrect response; **Omitted reactions**: a stimulus is regarded as omitted if the subject does not react within the stimulus presentation time or if the reaction made during that time is a delayed response to the preceding stimulus, and if he does not make a delayed response during the presentation of the subsequent item; **Median reaction time:** the reaction time (in seconds) is the time from the appearance of a stimulus to the pressing of a button on the response panel. The variable specifies the median of all correct (on-time and delayed) reaction times; **Number of stimuli:** this variable provides information on the number of stimuli presented. It is only calculated in Adaptive Mode. The number of stimuli serves as a control variable indicating how many stimuli were presented; **Reactions:** the variable Reactions is the total number of correct and incorrect responses – that is, the sum of all responses made.

SUPPLEMENTARY TABLE S2. Architecture of the two ANNs used in the regression task, detailing their layer types (type), number of neurons / fraction of the input units to drop for dense and dropout layers respectively (size) and activation functions, if used (activation).

	ANN1			ANN2		
**Layer**	**Type**	**Size**	**Activation**	**Type**	**Size**	**Activation**
1	Dense	5	ReLu	Dense	160	ReLu
2	Dense	5	ReLu	Dropout	0.2	
3	Dense	1	Linear	Dense	480	ReLu
4				Dropout	0.2	
5				Dense	256	ReLu
6				Dense	1	Linear

SUPPLEMENTARY TABLE S3. Weighted average **precision** of the classification models on predicting MFIS (total score) and MFIS (cognitive score) categorizations. The algorithms evaluated were Random Forest (RF), K-Nearest Neighbors (KNN), Support Vector Machine (SVM), Gaussian Naive Bayes (GNB), Complement Naive Bayes (CNB) and Logistic Regression (LR).

	**Classification type**	**RF**	**KNN**	**SVM**	**GNB**	**CNB**	**LR**	**Zero rule**
MFIS (total score)	Binary	0.68	0.68	0.68	0.68	0.88	0.68	0.68
	Three-classes	0.50	0.54	0.43	0.45	0.57	0.47	0.27
	Four-classes	0.23	0.16	0.21	0.22	0.36	0.18	0.09
MFIS (cognitive score)	Binary	0.75	0.74	0.76	0.74	0.71	0.76	0.76
	Three-classes	0.51	0.66	0.27	0.52	0.37	0.53	0.27
	Four-classes	0.17	0.23	0.29	0.24	0.33	0.35	0.15

SUPPLEMENTARY TABLE S4. Weighted average **recall** of the classification models on predicting MFIS (total score) and MFIS (cognitive score) categorizations. The algorithms evaluated were Random Forest (RF), K-Nearest Neighbors (KNN), Support Vector Machine (SVM), Gaussian Naive Bayes (GNB), Complement Naive Bayes (CNB) and Logistic Regression (LR).

	**Classification type**	**RF**	**KNN**	**SVM**	**GNB**	**CNB**	**LR**	**Zero rule**
MFIS (total score)	Binary	0.83	0.83	0.83	0.83	0.87	0.83	0.83
	Three-classes	0.57	0.52	0.52	0.52	0.57	0.57	0.52
	Four-classes	0.26	0.30	0.35	0.26	0.35	0.30	0.30
MFIS	Binary	0.83	0.74	0.87	0.74	0.57	0.87	0.87

(cognitive score)	Three-classes	0.57	0.65	0.52	0.52	0.39	0.57	0.52
	Four-classes	0.22	0.35	0.39	0.30	0.30	0.39	0.39
